# Institutional Housekeeping

**Published:** 1912-03-09

**Authors:** 


### INSTITUTIONAL HOUSEKEEPING.

**(Workers' Questions and Criticism of every kind will be welcomed.)**

### How to Avoid Wasting Bones.

Bones are not very promising articles for the housekeeper, but unless she deal warily with them, much precious nutritive matter will be lost to her establishment. Not only should none be thrown or given away, but the stock prepared from them should be nourishing and appetising. The amount of bone sent with meat must be strictly stipulated when meat is bought by contract, or otherwise a disproportionate amount will be sent.

After the dinner is served, there are always bones left on the dishes, for in the case of patients it is usual to cut the meat from the bone before serving, and this is an economical plan, as the bones can then be made use of. These bones when returned to the kitchen should be carefully looked over, scraps of fat and skin removed, portions of meat retained for made-up dishes, and the bones placed in the stock-pot. A jacketed stock-pot is the best, for it is easy to keep it at a steady temperature. Enough cold water should be used to cover the bones entirely, otherwise they are likely to decompose, and salt should be added. It requires at least five or six hours slow cooking to draw the nourishment from the bones and meat either cooked or uncooked. Vegetables may be added in large pieces and allowed to cook slowly in the stock; this improves the flavour. Stock thus prepared is most suitable for a foundation for soups and gravies, and very much adds to their palatability; it must not, however, be confounded with fresh-meat stock, which has much more strengthening and stimulating properties. All bones can thus be utilised, and soup prepared from this stock can be served to matron and house surgeons as required, and makes a pleasant variety twice a week, especially in winter, for the nurses' supper.

Tomatoes, split peas, lentils, haricot purées, artichokes, potatoes, mixed vegetables, carrots, barley, are some of the ingredients which can vary soups prepared from this stock; they can be thickened with sago, semolina, cornflour, or vermicelli, and if sufficiently good, served without additional flavouring. A bunch of sweet herbs, parsley, thyme, and marjoram, a little mace and three or four cloves also add much to the flavour of the stock.

The food value of stock made from cooked meat and bones is considerable, as the gelatine from the bones makes the stock easily digested and palatable, and meat juices are extracted from any scraps of meat added. Care however is needed in making stock from bones. It must cook slowly at simmering point, or the albumen contained in the meat is hardened and the flavour and nutritive properties are not extracted. In summer it is imperative that the stock should be boiled each day, as it is very liable to turn sour. Each night the liquid in the stock-pot must be strained and the bones removed.

The stock-pot should be taken to pieces, as far as possible, each day, and scalded thoroughly, for if the stock is allowed to get in the slightest degree sour, or to ferment, it is most unwholesome.

Raw bones sent with meat, for example in a boned round of beef, should be wiped carefully and the fat removed. In hot weather, if they are kept for more than a few hours, they should be baked for a few minutes on arrival. It is safer to scald or bake them before they are added to the hot liquid in the stock-pot, as fresh meat and bones if added to stock already cooking may turn it sour.

When the bones become full of small holes, this indicates that all the gelatinous substances and extractives have been boiled out.

Fat of any kind should not be placed in the stock-pot. Bones covered with sauce or any farinaceous substance should be washed, or the stock is very cloudy and quickly decomposes. Bones that have become sour should be burnt.

Thus treated, bones can be turned to very profitable account.

### Questions and Answers on Practical Points.

### Washing Dishes.

"Sanatorium Sister" inquires: I have to use very hot water for washing-up crockery, etc., used by the patients, and find it so painful to my hands, for the dish-cloths are nearly boiling. Is there any sort of simple appliance I could ask for to turn the things about?

There are various appliances for washing dirty dishes, but they are expensive and elaborate. Why not use a brush? Buy an English-made round brush, costing about 11d., such as is used for painting furniture. Soak it for twenty-four hours in cold water to swell the wood before use, for if this is not done the bristles will fall out. A brush is far more hygienic than mops or dish-cloths, for not only do its bristles penetrate into crevices of crockery, forks, etc., but much hotter water can be used. Moreover, it avoids the washing of greasy, unwholesome dish-cloths, which should be banished from every up-to-date kitchen. With a brush the right hand need never be put in the water, and articles can be easily pushed up out of the water to be grasped by the left hand.

### To Preserve Eggs.

"Thrifty": I want to be able to supply our Cottage Convalescent Home with eggs all the year round. I can get quantities of eggs in the spring and early summer very cheaply and wish to preserve them for the scarcity later on in the year. I have tried various methods—lime, salt, and fat—but without great success. What do you advise?

Lay them down in water-glass (silicate of soda). It can be bought from stores or chemists at about one shilling for a 4-lb. tin. Mix it as directed on the tin and lay the perfectly new-laid eggs in stone crocks, pouring the water-glass over them. This is by far the simplest and most efficacious method, and is highly successful.

